# Impaired mitochondrial homeostasis and neurodegeneration: towards new therapeutic targets?

**DOI:** 10.1007/s10863-014-9576-6

**Published:** 2014-09-13

**Authors:** Juan Carlos Corona, Michael R. Duchen

**Affiliations:** Department of Cell and Developmental Biology, University College London, London, WC1E 6BT UK

**Keywords:** Mitochondrial biogenesis, Autophagy, Permeability transition pore, Neuroprotection

## Abstract

The sustained integrity of the mitochondrial population of a cell is critical for maintained cell health, and disruption of that integrity is linked strongly to human disease, especially to the neurodegenerative diseases. These are appalling diseases causing untold levels of suffering for which treatment is woefully inadequate. Understanding the mechanisms that disturb mitochondrial homeostasis may therefore prove key to identification of potential new therapeutic pathways. Mechanisms causing mitochondrial dysfunction include the acute catastrophic loss of function caused by opening of the mitochondrial permeability transition pore (mPTP), which collapses bioenergetic function and initiates cell death. This is best characterised in ischaemic reperfusion injury, although it may also contribute to a number of other diseases. More insidious disturbances of mitochondrial homeostasis may result from impaired balance in the pathways that promote mitochondrial repair (biogenesis) and pathways that remove dysfunctional mitochondria (mitophagy). Impaired coordination between these processes is emerging as a key feature of a number of neurodegenerative and neuromuscular disorders. Here we review pathways that may prove to be valuable potential therapeutic targets, focussing on the molecular mechanisms that govern the coordination of these processes and their involvement in neurodegenerative diseases.

## Introduction

Mitochondria lie at the heart of energy provision for most cells and tissues, providing energy for the balance against negative entropy, for maintenance of cell structure and for active signalling. As mitochondria are crucial for the maintenance of cellular homeostasis, including ATP production and intracellular calcium regulation, and mitochondria regulate routes to cell death (reviewed in Osellame et al. 2012), it is hardly surprising that processes that disturb mitochondrial integrity lead to disease or to death.

Structurally, mitochondria are compartmentalised by two protein containing lipid bilayers: the external outer mitochondrial membrane (OMM), the inter membrane space (IMS) and the matrix, which is enclosed by the inner mitochondrial membrane (IMM) that is intensely but variably folded into cristae, which vastly expand the membrane surface area available for the bioenergetic transformations that are fundamental to mitochondrial function. The inner mitochondrial membrane houses the protein complexes of the electron transport chain, and provides a highly efficient and selective barrier to the flow of ions. The mitochondrial matrix contains the enzymes that drive the tricarboxylic acid cycle and beta-oxidation. The outer mitochondrial membrane has a less tightly regulated permeability, which allows the passage of low molecular weight substances, between the cytosol and the mitochondrial intermembrane space. The cross talk and transport of metabolites and proteins between the mitochondria and the rest of the cell are critical in mitochondrial biogenesis, in maintaining the competence of oxidative phosphorylation and production of ATP (reviewed in Kotiadis et al. 2014).

## Mitochondrial biogenesis

Mitochondrial biogenesis is the process by which cells generate new mitochondrial protein and, if required, increase mitochondrial mass. This is of necessity a complex and sophisticated process, as it requires the coordinated transcription and translation of two genomes, and so includes the import of nuclear encoded proteins, and the assembly of both mitochondrial and nuclear-derived products into an expanding mitochondrial reticulum, coupled to the recruitment of newly synthesized lipids (Dominy and Puigserver 2013; Zhu et al. 2013). At the molecular level, several transcription factors and cofactors are involved in the activation and regulation of mitochondrial biogenesis, in response to diverse stimuli, such as nutrient availability, hormones, growth factors and temperature fluctuations. These factors include: the ubiquitous transcription factors (SP1, YY1, CREB, MEF2/E-box), nuclear respiratory factors 1 and 2 (NRF1-2), REBOX/OXBOX, MT1 to 4 and estrogen-related receptors (ERRα, ERRβ, ERRγ) that work in concert with transcriptional coactivators of the peroxisome proliferator-activated receptor gamma coactivator 1α (PGC1α) family, (PGC1α, PGC1β and PGC1-related coactivator) (Dominy and Puigserver 2013; Puigserver et al. 1999; Scarpulla 2002).

PGC1α acts as a master regulator of mitochondrial biogenesis and energy metabolism and has the capability to integrate and coordinate the activity of multiple transcription factors, such as NRF1-2, PPARα and mitochondrial transcription factor A (mtTFA) (Dominy and Puigserver 2013; Puigserver et al. 1998). NRF1 and NFR2 govern the expression of multiple nuclear encoded mitochondrial proteins. For example, they regulate the transcription of mtTFA and transcription factor B proteins, which are in turn regulators of mitochondrial DNA transcription and replication (Dominy and Puigserver 2013; Gleyzer et al. 2005; Scarpulla 2008). Moreover, the transcriptional activity of NRF1 is linked to the expression of nuclear genes encoding subunits of the mitochondrial respiratory complexes, proteins related to mitochondrial import machinery, enzymes of haem biosynthesis, tRNA synthases and mitochondrial ribosomal proteins (Scarpulla 2008). Estrogen-related receptors (ERRα, ERRβ, ERRγ) are members of the nuclear hormone receptor family, and promote mitochondrial biogenesis in response to hormonal signals. ERRα regulates the transcription of nuclear genes encoding mitochondrial related factors, including proteins of the Krebs cycle, of mitochondrial fission and fusion, oxidative phosphorylation and of fatty acid oxidation (Dominy and Puigserver 2013).

Peroxisome proliferator-activated receptors (PPAR) are ligand activated transcription factors that belong to the nuclear hormone receptor superfamily, and are intimately involved in the regulation of mitochondrial biogenesis. The PPAR receptors include PPARα, PPARγ and PPARβ/δ. These pathways are subject to pharmacological control—PPARγ is activated by the thiazolidinediones, a group of drugs that regulate glucose metabolism, adipogenesis, differentiation, and the expression of several genes including antioxidant defences and inflammatory cytokines (Chung et al. 2008; Dello Russo et al. 2003; Jung et al. 2007; Nolan et al. 1994; Tontonoz et al. 1994). PPARγ agonists increase mitochondrial biogenesis, increasing the expression of PGC1α, NRF1-2, and mtTFA with a consequent increased mitochondrial DNA (mtDNA) copy number, increased oxygen consumption and an increased capacity for oxidative phosphorylation (Bogacka et al. 2005; Corona et al. 2014; Ghosh et al. 2007; Miglio et al. 2009; Strum et al. 2007).

## Mitochondrial biogenesis as target for neuroprotection

A number of studies have suggested potential roles for factors that regulate mitochondrial biogenesis in several neurodegenerative disorders. Thus, in mouse models of mitochondrial diseases the overexpression of PGC1α showed robust induction of mitochondrial biogenesis and increased the mitochondrial respiratory chain activity (Viscomi et al. 2011). Additionally, studies in human cells with complex III or IV deficiencies showed that increased expression of PGC1α, and/or its homologue PGC1β, improved mitochondrial respiration (Srivastava et al. 2009).

In cellular models of Parkinson’s disease (PD), the activation of PGC1α increased the expression of nuclear-encoded subunits of the mitochondrial respiratory chain and prevented the dopaminergic neuron loss induced by mutant α-synuclein or by the pesticide rotenone (Zheng et al. 2010). Recently it was shown that adenoviral delivery of PGC1α in the nigrostriatal system increased dopaminergic death (Ciron et al. 2012). This effect was explained by the excessive overexpression of PGC1α, resulting in mitochondrial hyperactivity and increased production of ROS. The overexpression of PGC1α in the SN was shown to reduce the expression of tyrosine hydroxylase (TH). The reduced TH expression was associated with loss of Pitx3, a transcription factor that is critical for the development and maintenance of dopaminergic cells. Expression of the neurotrophic factor BDNF, which is also regulated by Pitx3 was reduced. Therefore, the overexpression of PGC1α resulted in dopamine depletion associated with lower levels of Pitx3 and enhanced susceptibility to MPTP (Clark et al. 2012). Thus, studies of the role of PGC1α in relation to PD have provided inconsistent data.

While PGC1α is regulated at the transcriptional level, several post-translational modifications (phosphorylation, acetylation, SUMOylation, and methylation) (Puigserver and Spiegelman 2003; Rytinki and Palvimo 2009) and interactions may also be relevant for the maintenance of the metabolic competence of neurons. In that sense, and linking PGC1α biology to PD, it has been shown that the inactivation of DJ-1 (in which mutations associate with familial PD) causes decreased expression of human MnSOD. It seems that DJ-1 stimulates the activity of PGC1α, in turn influencing the transcription of MnSOD. Although DJ-1 does not interact with PGC1α directly, it inhibits the SUMOylation of a transcriptional repressor, pyrimidine tract-binding protein-associated splicing factor (PSF). PSF binds PGC1α and suppresses its transcriptional activity. SUMO-specific isopeptidase SENP-1 further enhances the synergy between DJ-1 and PGC1α, whereas a SUMO E3 ligase protein inhibitor of activated STAT Y completely blocks the synergy. Conversely, oxidative modification renders DJ-1 unable to inhibit SUMOylation, attenuating the transcriptional synergy between DJ-1 and PGC1α, and linking oxidative stress, DJ-1 dysfunction and PGC1α function, all associating with the pathophysiology of PD. Finally, the transcriptional dysregulation caused by the inactivation of DJ-1, either by pathogenic mutations or oxidation, could result in defective mitochondrial gene regulation that may contribute to the development of sporadic PD (Zhong and Xu 2008).

In a conditional knockout mouse with disruption of the gene for mtTFA, mtDNA expression and respiratory chain deficiency were reduced in dopaminergic neurons (Ekstrand et al. 2007). However, the overexpression of mtTFA and NRF1 in neuronal cells protected against MPP^+^-induced mitochondrial dysfunction in SHSY-5Y cells in culture (Piao et al. 2012). The PPARγ agonist rosiglitazone increased mitochondrial biogenesis, and increased oxygen consumption in sporadic and PINK1-associated PD cellular models (Corona et al. 2014).

Intriguingly, mitochondrial dysfunction, defective bioenergetics, striatal degeneration and a hyperkinetic movement disorder, features of Huntington’s disease (HD), were described in PGC1α knockout mice (Leone et al. 2005; Lin et al. 2004). The PGC1α knockout mice showed impaired motor performance, degeneration and an increased susceptibility to the mitochondrial complex II inhibitor, 3-nitropropionic acid, which alone causes a HD like illness in mice and is often used to model the disease. In similar vein, expression of PGC1α delivered into the striatum of HD transgenic mice using a lentiviral vector was neuroprotective (Cui et al. 2006). Indeed, overexpression of PGC1α enhanced the mitochondrial membrane potential and reduced mitochondrial toxicity in models of HD (Weydt et al. 2006). The PPARγ agonist rosiglitazone similarly increased mitochondrial biogenesis in striatal cells and protected against thapsigargin-induced mitochondrial dysfunction in mutant huntingtin-expressing cells (Quintanilla et al. 2008). In a transgenic mouse model of HD, treatment with rosiglitazone reduced huntingtin aggregates and normalized the expression of PGC1α, NRF1, NRF2 and mtTFA in the cortex. Also, rosiglitazone restored the reduced mitochondrial mass in mutant huntingtin expressing N2a cells (Chiang et al. 2012). The pan-PPAR agonist bezafibrate increased the numbers of muscle mitochondria, increased mitochondrial biogenesis and improved the phenotype, improved survival and reduced brain, muscle and BAT pathology in HD mice (Johri et al. 2012).

In the SOD1-G93A mouse model of familial amyotrophic lateral sclerosis (ALS), the elevation of PGC1α levels sustained mitochondrial biogenesis through end-stage disease and this activity was accompanied by retention of muscle function, delayed muscle atrophy, and significantly improved muscle endurance even at late stages of disease. However, survival was not extended (Da Cruz et al. 2012). In double transgenic mice overexpressing G93A mutant SOD1 and human mtTFA, the overexpression of mtTFA reduced indices of oxidative stress in motoneurons and delayed the onset of the disease in SOD1-G93A mice (Morimoto et al. 2012). Similarly, expression of PGC1α protected against mitochondrial fragmentation and reduced neuronal cell death in cultured motoneurons from SOD1-G93A rat and mouse models (Song et al. 2013).

**Fig. 1:**
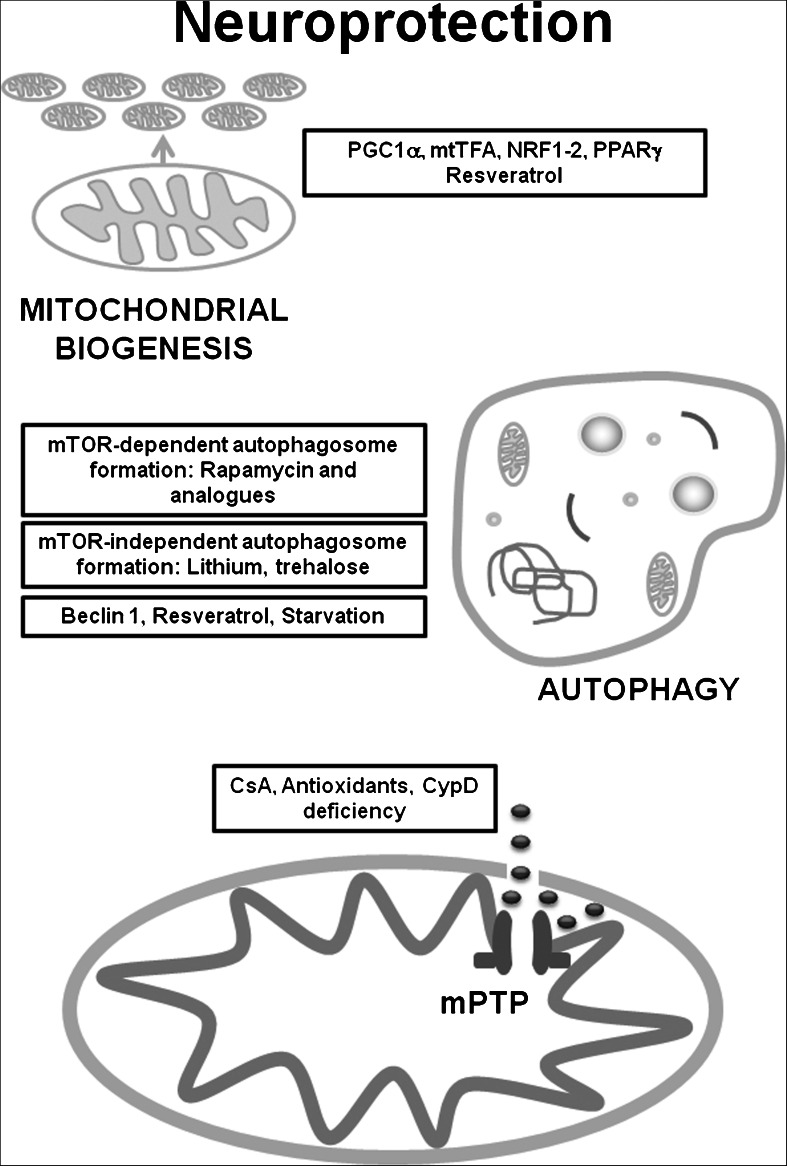
Mitochondrial biogenesis, autophagy and mPTP involved in neuroprotection. There are transcription factors that are intimately implicated in the regulation of mitochondrial biogenesis such as PGC1α, NRF1-2, mtTFA and PPARγ, also there are compounds like resveratrol that can stimulate mitochondrial biogenesis and protect against several neurodegenerative disorders. When autophagosome formation is impaired, mTOR-dependent and/or mTOR-independent autophagy upregulation may counteract neurodegeneration through the stimulation of autophagosome formation, also beclin1, starvation or compounds like resveratrol may stimulate autophagy. The mitochondrial channel mPTP is mainly associated with release of mitochondrial death signals and induction of apoptosis, therefore, mPTP inhibitors like CsA, antioxidants and the CypD deficiency, may be neuroprotective

In Alzheimer’s disease (AD) postmortem brain, PGC1α messenger RNA expression was significantly decreased, correlating with the progression of clinical dementia in the AD brain. Interestingly, PGC1α expression in primary cultures of cortico-hippocampal neurons derived from Tg2576 AD mice reversed glucose-induced amyloid-beta (Aβ) peptide production (Qin et al. 2009). PGC1α overexpression in N2a neuroblastoma cells also decreased secreted Aβ and increased the levels of non-amyloidogenic soluble Aβ (Katsouri et al. 2011). Furthermore, a genetic variant in mtTFA has been identified as a moderate risk factor for AD, suggesting that disturbed maintenance of mtDNA integrity or mitochondrial function may underlie neurodegeneration (Belin et al. 2007). Overexpression of mtTFA protected SH-SY5Y cells from Aβ-induced mitochondrial dysfunction (Xu et al. 2009). In both hippocampal tissues from mouse AD models and M17 cells expressing mutant amyloid precursor protein (APPswe), expression levels of PGC1α, NRF1, NRF2, and mtTFA were significantly decreased, suggesting reduced mitochondrial biogenesis. Nevertheless, overexpression of PGC1α exacerbated the impaired mitochondrial biogenesis and mitochondrial deficits in APPswe M17 cells (Sheng et al. 2012). The transcription factors implicated in the regulation of mitochondrial biogenesis are summarised in (Fig. 1).

## Autophagy

Autophagy is an evolutionarily conserved catabolic process that mediates the degradation of long-lived proteins and dysfunctional or superfluous organelles. Autophagy is induced by various adverse conditions such as limited nutrients, low oxygen levels, and decreased energy supply, and its action results in the release of degradation products, especially amino acids, back into the cytoplasm to be used in essential biosynthetic pathways (He and Klionsky 2009; Kundu and Thompson 2008; Levine and Kroemer 2008; Mizushima et al. 2008). The autophagic degradation routes are classified into three different pathways: macroautophagy, microautophagy, and chaperone-mediated autophagy (Massey et al. 2006; Mizushima 2009; Tolkovsky 2009). Macroautophagy is the major lysosomal pathway for the turnover of cytoplasmic components. During macroautophagy, portions of the cytoplasm are engulfed by a double-membrane phagophore that expands into a cytosolic vesicle called autophagosome; the completed autophagosome is targeted to the lysosome. The outer membrane of the autophagosome subsequently fuses with the lysosomal membrane, allowing hydrolases access to the inner autophagosome membrane and its cargo, which is degraded and recycled. Macroautophagy can mediate nonselective and bulk degradation of cytoplasmic contents, including entire organelles (Kundu and Thompson 2008). Finally, the autophagic load is degraded and the resulting essential biomolecules are recycled back to the cytoplasm to satisfy the anabolic and energetic requirements of the cell (Mizushima 2009). Among the three main types of autophagy, macroautophagy is the best characterized process and will hereafter be referred to as autophagy.

## Autophagy as a target for neuroprotection

In the CNS the accumulation of defective organelles and/or protein aggregates resulting from autophagic failure, may disturb axonal traffic and enhance neuronal toxicity (Nixon et al. 2008). Moreover, the autophagic machinery is required for the remodelling of neuronal dendrites and axons and hence for maintaining CNS plasticity (Fimia et al. 2007; Komatsu et al. 2007; Pickford et al. 2008; Wang et al. 2006). In neurons, the autophagic pathway and the ubiquitin proteasome system (UPS) are the major cellular routes for protein turnover. UPS-mediated degradation requires substrate proteins to unfold and pass through the narrow pore of the proteasomal barrel. As a result, autophagy is the main route for the degradation of toxic aggregate proteins, either in their oligomeric or in their aggregate forms. Therefore, dysregulation of autophagy has been proposed as a pathogenic mechanism contributing to several neurodegenerative disorders (Wong and Cuervo 2010).

In particular, there has been great excitement in recent years over possible roles of impaired autophagy in the pathophysiology of PD. In PD there is a long literature pointing towards defects in mitochondrial function, especially in relation to impaired complex I function (Schapira et al. 1990). This connection has been strengthened by the actions of toxins that target complex I (MPTP, rotenone) which cause a PD like neurodegeneration in animal models (Alam and Schmidt 2002; Betarbet et al. 2000; Corona et al. 2010). Further, whilst the occurrence of PD in man is predominantly sporadic and age related, a number of patients have familial forms of the disease. The systematic identification of mutations that increase the risk of PD, including genome wide association studies, has generated an ever-increasing list of proteins associated with PD (Bonifati 2012). Remarkably, a number of these lie on the pathways of autophagy. These include PINK1, that signals on the outer membrane of dysfunctional mitochondria to recruit the ubiquitin ligase Parkin, another PD related gene, and initiates removal of dysfunctional mitochondria by autophagy. Other PD associated proteins linked to the autophagy pathway include fbxo7 (Burchell et al. 2013), LRRK2 (Manzoni et al. 2013) and intriguingly the protein glucocerebrosidase (GBA), which lies on the lysosomal end of the pathway. Mutations of GBA give rise to the lysosomal storage disease, Gaucher disease, but mutations of GBA also carry the highest known risk of developing PD (Mazzulli et al. 2011; Sidransky et al. 2009). In a recent study (Osellame et al. 2013), we showed that in neurons cultured from the CNS of GBA knockout mice, autophagy was down regulated, mitochondrial function was profoundly compromised and there was early accumulation of α-synuclein, the major component of Lewy bodies, the pathological signature of PD which reflects also a failure of UPS mediated protein degradation pathways. Activities of respiratory complex I, II and III were impaired, mitochondria were fragmented, mass was increased but potential was decreased. Interestingly, the potential could be recovered after incubating the cells with MitoQ, an antioxidant targeted to complex I supporting a model whereby mitochondrial damage starts to accumulate as autophagic removal fails, leading to increased ROS generation, ROS mediated damage to the respiratory complexes, providing the basis for a destructive cycle that will lead to the progressive bioenergetic collapse culminating in cell death.

The pharmacological stimulation of autophagy has therefore been heralded as a novel and promising clinical strategy for the treatment of some neurodegenerative disorders. The mammalian target of rapamycin (mTOR), a master regulator of nutrient and growth factor signalling, is one of the critical components involved in controlling the induction of autophagy (He and Klionsky 2009). In most cell types, mTOR activity is necessary and sufficient to suppress autophagy under favourable growth conditions. In fact, it has been reported that inhibition of mTOR with rapamycin induced autophagy and reduced toxicity of polyglutamine expansions in fly and mouse models of HD (Ravikumar et al. 2004). There are other chemical agents capable of inducing autophagy in an mTOR-independent manner. In that sense, lithium treatment induced mTOR-independent autophagy by inhibiting inositol monophosphatase and protected against neurodegeneration in an HD fly model (Sarkar et al. 2008). The mTOR independent autophagy inducer trehalose alleviated polyglutamine-mediated pathology in a mouse model of HD (Tanaka et al. 2004). Other studies have shown that elevation of the lysosomal proteases accelerated clearance of mutant huntingtin from HEK cells through an autophagic mechanism (Liang et al. 2011).

Several in vitro studies have demonstrated that induction of autophagy with different compounds such as resveratrol (Wu et al. 2011), kaempferol (Filomeni et al. 2012) and Isorhynchophylline (Lu et al. 2012), resulted in improvements in different models of PD. Also, beclin 1 (a regulator of the autophagy pathway) gene transfer activated autophagy and ameliorated the neurodegenerative pathology in α-synuclein models of PD/Lewy body diseases (Spencer et al. 2009). The administration of a lentiviral vector expressing beclin 1 reduced both intracellular and extracellular Aβ pathology in APP transgenic mice model of AD (Pickford et al. 2008). Moreover, resveratrol reduced neurodegeneration in the hippocampus and prevented learning impairment in a model of AD (Kim et al. 2007). In SOD1-G93A mice, daily administration of lithium delayed ALS progression and the neuroprotection was accompanied by the activation of autophagy in motoneurons (Fornai et al. 2008). Upregulation of autophagic genes displayed similar results in Caenorhabditis elegans model of ALS (Li et al. 2013). The molecular chaperone trehalose (Gomes et al. 2010) and resveratrol (Kim et al. 2007) have also shown protection against neurodegeneration in models of ALS. Food restriction or starvation, another established autophagy inducer, protected the spinal cord of SOD1-G93A mice, only at onset stage but not at pre-end stage of ALS (Zhang et al. 2013). However, rapamycin augmented motoneuron degeneration in the SOD1-G93A mice (Zhang et al. 2011). The factors implicated in the stimulation of autophagy are summarised in (Fig. 1).

## mPTP

Opening of the mitochondrial permeability transition pore (mPTP) mediates a catastrophic increase in the permeability of the inner mitochondrial membrane that collapses the potential, preventing ATP synthesis and leading to cell death. Its molecular identity is controversial. There has been a broad consensus that the pore represents a conformational change of components of the inner mitochondrial membrane that have another function in normal respiring mitochondria. Currently, the favoured candidate is the F1Fo-ATP synthase (Alavian et al. 2014; Giorgio et al. 2013). Currently, the only certain component of the pore complex is cyclophilin D, (CypD) which acts as a regulator of pore opening. The involvement of CypD is crucial, as it renders the pore accessible to pharmacological intervention through cyclosporin A (CsA) and related compounds.

The opening of the mPTP leads to a transition in permeability of the inner membrane from extremely low to freely permeable to solutes up to 1.5 kDa. The mPTP can be opened in cells and respiring isolated mitochondria by Ca^2+^ overload and by pro-oxidants, modulated by adenine nucleotides and by phosphate (Gunter and Pfeiffer 1990; Halestrap 2009). The open mPTP may be closed by removal of Ca^2+^ with EGTA or the addition of ADP, Mg^2+^, or CsA (Bernardi and von Stockum 2012; Crompton et al. 1988; Dube et al. 2012). The opening of mPTP is classically initiated by an increase in matrix Ca^2+^ in the presence of phosphate, while depletion of adenine nucleotides, oxidative stress or a decrease in mitochondrial potential lower the threshold for Ca^2+^ dependent opening of the pore or mitochondria are depolarized by FCCP (Crompton and Costi 1990; Scorrano et al. 1997). Moreover, higher Ca^2+^ concentrations are needed for pore opening in the presence of inhibitors including high negative membrane potential, low matrix pH, ADP, Sr and Mg^2+^ (Bernardi and von Stockum 2012; Kinnally et al. 2011). The rapid removal of Ca^2+^ from the cytosol through mitochondrial Ca^2+^ uptake and its subsequent release from mitochondria through mPTP flickering could prevent Ca^2+^-inactivation of plasma membrane channels essential to refilling Ca^2+^ stores while allowing signalling in these microdomains (Pastorino et al. 1999).

Mitochondrial Ca^2+^ overload leads to the induction of the mPTP, resulting in osmotic swelling and a collapse of the mitochondrial membrane potential. This will cause complete bioenergetic collapse and will lead inevitably to necrotic cell death in most cell types. In cells with a high glycolytic capacity, the swelling, the resulting rupture of the outer membrane and the release of pro-apoptotic factors into the cytosol may initiate apoptotic cell death (Kinnally et al. 2011; Ryu et al. 2010). The specific role of the mPTP in apoptotic cell death has been somewhat controversial—thus knockout of CypD was protective against necrotic but not apoptotic cell death (Li et al. 2004; Nakagawa et al. 2005).

mPTP opening is different in liver and brain mitochondria, as the liver mitochondria have lower Ca^2+^ capacity than brain mitochondria (Panov et al. 2007). The brain mitochondria are less sensitive to the damaging effect of Ca^2+^ and are less sensitive to the protective effect of CsA during Ca^2+^ load. The osmotic swelling of the brain mitochondria is rather small in comparison with liver mitochondria. Interestingly, liver and neuronal cells have different strategies towards the Ca^2+^-induced mPTP and ROS generation, the observed differences in the Ca^2+^-induced mPTP and ROS generation may be due to the different organs and species used experimentally (Panov et al. 2007). Therefore, the differences in mPTP properties in diverse tissues might be caused by different proteins/isoforms that participate in the function/regulation of pore opening.

## mPTP as a target for neuroprotection

Once open, the mPTP collapses the mitochondrial membrane potential, oxidative phosphorylation ceases and the ATPase may even run in reverse accelerating ATP consumption and driving necrotic cell death (Campanella et al. 2009). However, mPTP opening also induces mitochondrial swelling and rupture of the outer membrane leading to the release of pro-apoptotic proteins, including cytochrome *c* and apoptosis-inducing factor, from the mitochondrial intermembrane space into the cytoplasm (Hengartner 2000; Hirsch et al. 1997; Martinou and Green 2001). As a consequence, mPTP induction may progress to apoptotic cell death (Fiskum 2000; Hirsch et al. 1997), although this will depend crucially on the glycolytic capacity of the cell as apoptosis is ATP dependent. Neuroprotection by CsA, the canonical inhibitor of the mPTP, is most clearly established in the context of stroke—i.e. ischemia-induced brain injury (Shiga et al. 1992). The mechanism of this protection is ambiguous as CsA also inhibits calcineurin, which is also implicated in ischaemic neuronal injury, but cerebral infarct size is reduced in CypD knockout mice (Wang et al. 2009) lending support to a specific role of the mPTP in stroke. In the heart, a role for the mPTP in injury following ischaemia and reperfusion is sufficiently compelling to support clinical trials (Piot et al. 2008). Sanglifehrin-A, another, compound which inhibits mPTP opening, protected myocytes from ischaemia-reperfusion injury (Hausenloy et al. 2003). We are not aware of studies using this compound in stroke models.

The mPTP has also been implicated in AD pathophysiology, as exposure of neurons to Aβ peptides lower the threshold for mPTP induction (Moreira et al. 2001). The Aβ exposure caused the progressive loss of mitochondrial membrane potential in astrocytes, accompanied by transient mitochondrial depolarisations caused by reversible openings of the mPTP driven by sporadic calcium signals combined with oxidative stress driven by activation of the NADPH oxidase. The transient mitochondrial depolarisatons were inhibited by CsA and were absent in cultures from CypD knockout mice (Abeti et al. 2011; Abramov et al. 2004a, 2004b, 2007). CypD deficiency improved learning and memory and synaptic function in an AD mouse model and alleviated Aβ-mediated reductions in long term potentiation (Du et al. 2008). Also, disease onset and life expectancy of G93A-SOD1 mice (a model of ALS) were significantly increased in animals in which CypD was knocked out. The effect of CypD deletion was much more prominent in females than in males (Martin et al. 2009).

Recently, it was shown that the loss of DJ1 (a gene linked to recessively inherited forms of PD), increased the rate of ROS production, associated with a reduced mitochondrial transmembrane potential and increased probability of mPTP opening (Giaime et al. 2012), all the changes were restored by antioxidant treatment. Similarly, the loss of PINK1 increased mitochondrial Ca^2+^ and increased the probability of mPTP opening (Gautier et al. 2012). Our own work suggested that PINK1 knockout caused impaired mitochondrial sodium calcium exchange activity leading to mitochondrial calcium overload and mPTP opening (Gandhi et al. 2009). It is not at all clear how these roles for PINK1 match with its apparent role in driving mitochondrial autophagy. Dopamine itself seems to cause oxidative stress in dopaminergic neurons, and exposure of PINK1 knockout cells to dopamine led to mitochondrial depolarisation caused by mPTP opening. The dopamine-induced mPTP opening, and dopamine-induced cell death, were prevented by inhibition of ROS production, by provision of respiratory chain substrates, and by reducing Ca^2+^ signals (Gandhi et al. 2012).

A reduction of the oxidation of critical thiol groups of the mPTP, which sensitize pore opening to Ca^2+^, represents a mechanism involved in preconditioning-mediated inhibition of mPTP opening (Halestrap et al. 2007). In a recent study, it was demonstrated that the treatment of mutant huntingtin-expressing cells (STHdh^Q111/Q111^) and cortical neurons expressing mutant huntingtin, with thapsigargin (a SERCA antagonist that releases ER Ca^2+^, raising cytosolic [Ca^2+^] levels), decreased mitochondrial membrane potential and caused cell death. The data suggested that thapsigargin induced opening of mPTP in striatal cells expressing mutant huntingtin, as the effects were prevented by pre-treatment with CsA (Quintanilla et al. 2013). These findings are consistent with the observations that CAG (polyglutamine repeats) sensitise mitochondria to Ca^2+^ induced depolarisation (Panov et al. 2002) and support a possible role for the pore in the pathophysiology of HD. The factors related to mPTP implicated in neuroprotection are summarised in (Fig. 1).

## Conclusion

Accumulating evidence has pointed to a major role for mitochondrial dysfunction in many of the major neurodegenerative disorders. In some cases mitochondrial dysfunction seems a primary mechanism driving the pathophysiology, whilst in others the mitochondria lie on the pathways to cell death as targets of other primary pathologies. In either case, understanding the mechanisms that converge on mitochondria may help define novel therapeutic strategies for these otherwise largely intractable appalling diseases.

Given that mitochondrial dysfunction is an early pathological feature of several neurodegenerative diseases, targeting impaired pathways of mitochondrial homeostasis may represent a powerful therapeutic strategy for treatment of the diseases in their early stages. Mitochondrial dysfunction may increase ROS generation, cause abnormal protein–protein interactions, and of course may compromise cellular bioenergetic integrity or lead to cell death. Therefore, therapeutic approaches focussing primarily on mitochondrial homeostasis may improve mitochondrial function and ameliorate the damage in neurodegenerative diseases. Finally, understanding the molecular mechanisms will bring new insights into the roles of mitochondrial homeostasis in normal and pathological conditions and could provide new therapeutic alternatives.

## References

[CR1] Abeti R, Abramov AY, Duchen MR (2011). Beta-amyloid activates PARP causing astrocytic metabolic failure and neuronal death. Brain.

[CR2] Abramov AY, Canevari L, Duchen MR (2004). Beta-amyloid peptides induce mitochondrial dysfunction and oxidative stress in astrocytes and death of neurons through activation of NADPH oxidase. J Neurosci.

[CR3] Abramov AY, Canevari L, Duchen MR (2004). Calcium signals induced by amyloid beta peptide and their consequences in neurons and astrocytes in culture. Biochim Biophys Acta.

[CR4] Abramov AY, Fraley C, Diao CT, Winkfein R, Colicos MA, Duchen MR (2007). Targeted polyphosphatase expression alters mitochondrial metabolism and inhibits calcium-dependent cell death. Proc Natl Acad Sci U S A.

[CR5] Alam M, Schmidt WJ (2002). Rotenone destroys dopaminergic neurons and induces parkinsonian symptoms in rats. Behav Brain Res.

[CR6] Alavian KN, Beutner G, Lazrove E, Sacchetti S, Park HA, Licznerski P (2014). An uncoupling channel within the c-subunit ring of the F1FO ATP synthase is the mitochondrial permeability transition pore. Proc Natl Acad Sci U S A.

[CR7] Belin AC, Bjork BF, Westerlund M, Galter D, Sydow O, Lind C (2007). Association study of two genetic variants in mitochondrial transcription factor A (TFAM) in Alzheimer’s and Parkinson’s disease. Neurosci Lett.

[CR8] Bernardi P, von Stockum S (2012). The permeability transition pore as a Ca(2+) release channel: new answers to an old question. Cell Calcium.

[CR9] Betarbet R, Sherer TB, MacKenzie G, Garcia-Osuna M, Panov AV, Greenamyre JT (2000). Chronic systemic pesticide exposure reproduces features of Parkinson’s disease. Nat Neurosci.

[CR10] Bogacka I, Xie H, Bray GA, Smith SR (2005). Pioglitazone induces mitochondrial biogenesis in human subcutaneous adipose tissue in vivo. Diabetes.

[CR11] Bonifati V (2012). Autosomal recessive parkinsonism. Parkinsonism Relat Disord.

[CR12] Burchell VS, Nelson DE, Sanchez-Martinez A, Delgado-Camprubi M, Ivatt RM, Pogson JH (2013). The Parkinson’s disease-linked proteins Fbxo7 and Parkin interact to mediate mitophagy. Nat Neurosci.

[CR13] Campanella M, Parker N, Tan CH, Hall AM, Duchen MR (2009). IF(1): setting the pace of the F(1)F(o)-ATP synthase. Trends Biochem Sci.

[CR14] Chiang MC, Chern Y, Huang RN (2012). PPARgamma rescue of the mitochondrial dysfunction in Huntington’s disease. Neurobiol Dis.

[CR15] Chung JH, Seo AY, Chung SW, Kim MK, Leeuwenburgh C, Yu BP (2008). Molecular mechanism of PPAR in the regulation of age-related inflammation. Ageing Res Rev.

[CR16] Ciron C, Lengacher S, Dusonchet J, Aebischer P, Schneider BL (2012). Sustained expression of PGC-1alpha in the rat nigrostriatal system selectively impairs dopaminergic function. Hum Mol Genet.

[CR17] Clark J, Silvaggi JM, Kiselak T, Zheng K, Clore EL, Dai Y (2012). Pgc-1alpha overexpression downregulates Pitx3 and increases susceptibility to MPTP toxicity associated with decreased Bdnf. PLoS One.

[CR18] Corona JC, Gimenez-Cassina A, Lim F, Diaz-Nido J (2010). Hexokinase II gene transfer protects against neurodegeneration in the rotenone and MPTP mouse models of Parkinson’s disease. J Neurosci Res.

[CR19] Corona JC, de Souza SC, Duchen MR (2014). PPARgamma activation rescues mitochondrial function from inhibition of complex I and loss of PINK1. Exp Neurol.

[CR20] Crompton M, Costi A (1990). A heart mitochondrial Ca2(+)-dependent pore of possible relevance to re-perfusion-induced injury. Evidence that ADP facilitates pore interconversion between the closed and open states. Biochem J.

[CR21] Crompton M, Ellinger H, Costi A (1988). Inhibition by cyclosporin A of a Ca2+-dependent pore in heart mitochondria activated by inorganic phosphate and oxidative stress. Biochem J.

[CR22] Cui L, Jeong H, Borovecki F, Parkhurst CN, Tanese N, Krainc D (2006). Transcriptional repression of PGC-1alpha by mutant huntingtin leads to mitochondrial dysfunction and neurodegeneration. Cell.

[CR23] Da Cruz S, Parone PA, Lopes VS, Lillo C, McAlonis-Downes M, Lee SK (2012). Elevated PGC-1alpha activity sustains mitochondrial biogenesis and muscle function without extending survival in a mouse model of inherited ALS. Cell Metab.

[CR24] Dello Russo C, Gavrilyuk V, Weinberg G, Almeida A, Bolanos JP, Palmer J (2003). Peroxisome proliferator-activated receptor gamma thiazolidinedione agonists increase glucose metabolism in astrocytes. J Biol Chem.

[CR25] Dominy JE, Puigserver P (2013) Mitochondrial biogenesis through activation of nuclear signaling proteins. Cold Spring Harb Perspect Biol 5 10.1101/cshperspect.a015008 PMC3685894 23818499

[CR26] Du H, Guo L, Fang F, Chen D, Sosunov AA, McKhann GM (2008). Cyclophilin D deficiency attenuates mitochondrial and neuronal perturbation and ameliorates learning and memory in Alzheimer’s disease. Nat Med.

[CR27] Dube H, Selwood D, Malouitre S, Capano M, Simone MI, Crompton M (2012). A mitochondrial-targeted cyclosporin A with high binding affinity for cyclophilin D yields improved cytoprotection of cardiomyocytes. Biochem J.

[CR28] Ekstrand MI, Terzioglu M, Galter D, Zhu S, Hofstetter C, Lindqvist E (2007). Progressive parkinsonism in mice with respiratory-chain-deficient dopamine neurons. Proc Natl Acad Sci U S A.

[CR29] Filomeni G, Graziani I, De Zio D, Dini L, Centonze D, Rotilio G (2012). Neuroprotection of kaempferol by autophagy in models of rotenone-mediated acute toxicity: possible implications for Parkinson’s disease. Neurobiol Aging.

[CR30] Fimia GM, Stoykova A, Romagnoli A, Giunta L, Di Bartolomeo S, Nardacci R (2007). Ambra1 regulates autophagy and development of the nervous system. Nature.

[CR31] Fiskum G (2000). Mitochondrial participation in ischemic and traumatic neural cell death. J Neurotrauma.

[CR32] Fornai F, Longone P, Cafaro L, Kastsiuchenka O, Ferrucci M, Manca ML (2008). Lithium delays progression of amyotrophic lateral sclerosis. Proc Natl Acad Sci U S A.

[CR33] Gandhi S, Wood-Kaczmar A, Yao Z, Plun-Favreau H, Deas E, Klupsch K (2009). PINK1-associated Parkinson’s disease is caused by neuronal vulnerability to calcium-induced cell death. Mol Cell.

[CR34] Gandhi S, Vaarmann A, Yao Z, Duchen MR, Wood NW, Abramov AY (2012). Dopamine induced neurodegeneration in a PINK1 model of Parkinson’s disease. PLoS One.

[CR35] Gautier CA, Giaime E, Caballero E, Nunez L, Song Z, Chan D (2012). Regulation of mitochondrial permeability transition pore by PINK1. Mol Neurodegener.

[CR36] Ghosh S, Patel N, Rahn D, McAllister J, Sadeghi S, Horwitz G (2007). The thiazolidinedione pioglitazone alters mitochondrial function in human neuron-like cells. Mol Pharmacol.

[CR37] Giaime E, Yamaguchi H, Gautier CA, Kitada T, Shen J (2012). Loss of DJ-1 does not affect mitochondrial respiration but increases ROS production and mitochondrial permeability transition pore opening. PLoS One.

[CR38] Giorgio V, von Stockum S, Antoniel M, Fabbro A, Fogolari F, Forte M (2013). Dimers of mitochondrial ATP synthase form the permeability transition pore. Proc Natl Acad Sci U S A.

[CR39] Gleyzer N, Vercauteren K, Scarpulla RC (2005). Control of mitochondrial transcription specificity factors (TFB1M and TFB2M) by nuclear respiratory factors (NRF-1 and NRF-2) and PGC-1 family coactivators. Mol Cell Biol.

[CR40] Gomes C, Escrevente C, Costa J (2010). Mutant superoxide dismutase 1 overexpression in NSC-34 cells: effect of trehalose on aggregation, TDP-43 localization and levels of co-expressed glycoproteins. Neurosci Lett.

[CR41] Gunter TE, Pfeiffer DR (1990). Mechanisms by which mitochondria transport calcium. Am J Physiol.

[CR42] Halestrap AP (2009). What is the mitochondrial permeability transition pore?. J Mol Cell Cardiol.

[CR43] Halestrap AP, Clarke SJ, Khaliulin I (2007). The role of mitochondria in protection of the heart by preconditioning. Biochim Biophys Acta.

[CR44] Hausenloy DJ, Duchen MR, Yellon DM (2003). Inhibiting mitochondrial permeability transition pore opening at reperfusion protects against ischaemia-reperfusion injury. Cardiovasc Res.

[CR45] He C, Klionsky DJ (2009). Regulation mechanisms and signaling pathways of autophagy. Annu Rev Genet.

[CR46] Hengartner MO (2000). The biochemistry of apoptosis. Nature.

[CR47] Hirsch T, Marzo I, Kroemer G (1997). Role of the mitochondrial permeability transition pore in apoptosis. Biosci Rep.

[CR48] Johri A, Calingasan NY, Hennessey TM, Sharma A, Yang L, Wille E (2012). Pharmacologic activation of mitochondrial biogenesis exerts widespread beneficial effects in a transgenic mouse model of Huntington’s disease. Hum Mol Genet.

[CR49] Jung TW, Lee JY, Shim WS, Kang ES, Kim SK, Ahn CW (2007). Rosiglitazone protects human neuroblastoma SH-SY5Y cells against MPP+ induced cytotoxicity via inhibition of mitochondrial dysfunction and ROS production. J Neurol Sci.

[CR50] Katsouri L, Parr C, Bogdanovic N, Willem M, Sastre M (2011). PPARgamma co-activator-1alpha (PGC-1alpha) reduces amyloid-beta generation through a PPARgamma-dependent mechanism. J Alzheimers Dis.

[CR51] Kim D, Nguyen MD, Dobbin MM, Fischer A, Sananbenesi F, Rodgers JT (2007). SIRT1 deacetylase protects against neurodegeneration in models for Alzheimer’s disease and amyotrophic lateral sclerosis. EMBO J.

[CR52] Kinnally KW, Peixoto PM, Ryu SY, Dejean LM (2011). Is mPTP the gatekeeper for necrosis, apoptosis, or both?. Biochim Biophys Acta.

[CR53] Komatsu M, Ueno T, Waguri S, Uchiyama Y, Kominami E, Tanaka K (2007). Constitutive autophagy: vital role in clearance of unfavorable proteins in neurons. Cell Death Differ.

[CR54] Kotiadis VN, Duchen MR, Osellame LD (2014). Mitochondrial quality control and communications with the nucleus are important in maintaining mitochondrial function and cell health. Biochim Biophys Acta.

[CR55] Kundu M, Thompson CB (2008). Autophagy: basic principles and relevance to disease. Annu Rev Pathol.

[CR56] Leone TC, Lehman JJ, Finck BN, Schaeffer PJ, Wende AR, Boudina S (2005). PGC-1alpha deficiency causes multi-system energy metabolic derangements: muscle dysfunction, abnormal weight control and hepatic steatosis. PLoS Biol.

[CR57] Levine B, Kroemer G (2008). Autophagy in the pathogenesis of disease. Cell.

[CR58] Li Y, Johnson N, Capano M, Edwards M, Crompton M (2004). Cyclophilin-D promotes the mitochondrial permeability transition but has opposite effects on apoptosis and necrosis. Biochem J.

[CR59] Li J, Huang KX, Le WD (2013). Establishing a novel C. elegans model to investigate the role of autophagy in amyotrophic lateral sclerosis. Acta Pharmacol Sin.

[CR60] Liang Q, Ouyang X, Schneider L, Zhang J (2011). Reduction of mutant huntingtin accumulation and toxicity by lysosomal cathepsins D and B in neurons. Mol Neurodegener.

[CR61] Lin J, Wu PH, Tarr PT, Lindenberg KS, St-Pierre J, Zhang CY (2004). Defects in adaptive energy metabolism with CNS-linked hyperactivity in PGC-1alpha null mice. Cell.

[CR62] Lu JH, Tan JQ, Durairajan SS, Liu LF, Zhang ZH, Ma L (2012). Isorhynchophylline, a natural alkaloid, promotes the degradation of alpha-synuclein in neuronal cells via inducing autophagy. Autophagy.

[CR63] Manzoni C, Mamais A, Dihanich S, Abeti R, Soutar MP, Plun-Favreau H (2013). Inhibition of LRRK2 kinase activity stimulates macroautophagy. Biochim Biophys Acta.

[CR64] Martin LJ, Gertz B, Pan Y, Price AC, Molkentin JD, Chang Q (2009). The mitochondrial permeability transition pore in motor neurons: involvement in the pathobiology of ALS mice. Exp Neurol.

[CR65] Martinou JC, Green DR (2001). Breaking the mitochondrial barrier. Nat Rev Mol Cell Biol.

[CR66] Massey AC, Kaushik S, Sovak G, Kiffin R, Cuervo AM (2006). Consequences of the selective blockage of chaperone-mediated autophagy. Proc Natl Acad Sci U S A.

[CR67] Mazzulli JR, Xu YH, Sun Y, Knight AL, McLean PJ, Caldwell GA (2011). Gaucher disease glucocerebrosidase and alpha-synuclein form a bidirectional pathogenic loop in synucleinopathies. Cell.

[CR68] Miglio G, Rosa AC, Rattazzi L, Collino M, Lombardi G, Fantozzi R (2009). PPARgamma stimulation promotes mitochondrial biogenesis and prevents glucose deprivation-induced neuronal cell loss. Neurochem Int.

[CR69] Mizushima N (2009). Physiological functions of autophagy. Curr Top Microbiol Immunol.

[CR70] Mizushima N, Levine B, Cuervo AM, Klionsky DJ (2008). Autophagy fights disease through cellular self-digestion. Nature.

[CR71] Moreira PI, Santos MS, Moreno A, Oliveira C (2001). Amyloid beta-peptide promotes permeability transition pore in brain mitochondria. Biosci Rep.

[CR72] Morimoto N, Miyazaki K, Kurata T, Ikeda Y, Matsuura T, Kang D (2012). Effect of mitochondrial transcription factor a overexpression on motor neurons in amyotrophic lateral sclerosis model mice. J Neurosci Res.

[CR73] Nakagawa T, Shimizu S, Watanabe T, Yamaguchi O, Otsu K, Yamagata H (2005). Cyclophilin D-dependent mitochondrial permeability transition regulates some necrotic but not apoptotic cell death. Nature.

[CR74] Nixon RA, Yang DS, Lee JH (2008). Neurodegenerative lysosomal disorders: a continuum from development to late age. Autophagy.

[CR75] Nolan JJ, Ludvik B, Beerdsen P, Joyce M, Olefsky J (1994). Improvement in glucose tolerance and insulin resistance in obese subjects treated with troglitazone. N Engl J Med.

[CR76] Osellame LD, Blacker TS, Duchen MR (2012). Cellular and molecular mechanisms of mitochondrial function. Best Pract Res Clin Endocrinol Metab.

[CR77] Osellame LD, Rahim AA, Hargreaves IP, Gegg ME, Richard-Londt A, Brandner S (2013). Mitochondria and quality control defects in a mouse model of Gaucher disease–links to Parkinson’s disease. Cell Metab.

[CR78] Panov AV, Gutekunst CA, Leavitt BR, Hayden MR, Burke JR, Strittmatter WJ (2002). Early mitochondrial calcium defects in Huntington’s disease are a direct effect of polyglutamines. Nat Neurosci.

[CR79] Panov A, Dikalov S, Shalbuyeva N, Hemendinger R, Greenamyre JT, Rosenfeld J (2007). Species- and tissue-specific relationships between mitochondrial permeability transition and generation of ROS in brain and liver mitochondria of rats and mice. Am J Physiol Cell Physiol.

[CR80] Pastorino JG, Tafani M, Rothman RJ, Marcinkeviciute A, Hoek JB, Farber JL (1999). Functional consequences of the sustained or transient activation by Bax of the mitochondrial permeability transition pore. J Biol Chem.

[CR81] Piao Y, Kim HG, Oh MS, Pak YK (2012). Overexpression of TFAM, NRF-1 and myr-AKT protects the MPP(+)-induced mitochondrial dysfunctions in neuronal cells. Biochim Biophys Acta.

[CR82] Pickford F, Masliah E, Britschgi M, Lucin K, Narasimhan R, Jaeger PA (2008). The autophagy-related protein beclin 1 shows reduced expression in early Alzheimer disease and regulates amyloid beta accumulation in mice. J Clin Invest.

[CR83] Piot C, Croisille P, Staat P, Thibault H, Rioufol G, Mewton N (2008). Effect of cyclosporine on reperfusion injury in acute myocardial infarction. N Engl J Med.

[CR84] Puigserver P, Spiegelman BM (2003). Peroxisome proliferator-activated receptor-gamma coactivator 1 alpha (PGC-1 alpha): transcriptional coactivator and metabolic regulator. Endocr Rev.

[CR85] Puigserver P, Wu Z, Park CW, Graves R, Wright M, Spiegelman BM (1998). A cold-inducible coactivator of nuclear receptors linked to adaptive thermogenesis. Cell.

[CR86] Puigserver P, Adelmant G, Wu Z, Fan M, Xu J, O’Malley B (1999). Activation of PPARgamma coactivator-1 through transcription factor docking. Science.

[CR87] Qin W, Haroutunian V, Katsel P, Cardozo CP, Ho L, Buxbaum JD (2009). PGC-1alpha expression decreases in the Alzheimer disease brain as a function of dementia. Arch Neurol.

[CR88] Quintanilla RA, Jin YN, Fuenzalida K, Bronfman M, Johnson GV (2008). Rosiglitazone treatment prevents mitochondrial dysfunction in mutant huntingtin-expressing cells: possible role of peroxisome proliferator-activated receptor-gamma (PPARgamma) in the pathogenesis of Huntington disease. J Biol Chem.

[CR89] Quintanilla RA, Jin YN, von Bernhardi R, Johnson GV (2013). Mitochondrial permeability transition pore induces mitochondria injury in Huntington disease. Mol Neurodegener.

[CR90] Ravikumar B, Vacher C, Berger Z, Davies JE, Luo S, Oroz LG (2004). Inhibition of mTOR induces autophagy and reduces toxicity of polyglutamine expansions in fly and mouse models of Huntington disease. Nat Genet.

[CR91] Rytinki MM, Palvimo JJ (2009). SUMOylation attenuates the function of PGC-1alpha. J Biol Chem.

[CR92] Ryu SY, Peixoto PM, Teijido O, Dejean LM, Kinnally KW (2010). Role of mitochondrial ion channels in cell death. Biofactors.

[CR93] Sarkar S, Krishna G, Imarisio S, Saiki S, O’Kane CJ, Rubinsztein DC (2008). A rational mechanism for combination treatment of Huntington’s disease using lithium and rapamycin. Hum Mol Genet.

[CR94] Scarpulla RC (2002). Nuclear activators and coactivators in mammalian mitochondrial biogenesis. Biochim Biophys Acta.

[CR95] Scarpulla RC (2008). Transcriptional paradigms in mammalian mitochondrial biogenesis and function. Physiol Rev.

[CR96] Schapira AH, Cooper JM, Dexter D, Clark JB, Jenner P, Marsden CD (1990). Mitochondrial complex I deficiency in Parkinson’s disease. J Neurochem.

[CR97] Scorrano L, Petronilli V, Bernardi P (1997). On the voltage dependence of the mitochondrial permeability transition pore. A critical appraisal. J Biol Chem.

[CR98] Sheng B, Wang X, Su B, Lee HG, Casadesus G, Perry G (2012). Impaired mitochondrial biogenesis contributes to mitochondrial dysfunction in Alzheimer’s disease. J Neurochem.

[CR99] Shiga Y, Onodera H, Matsuo Y, Kogure K (1992). Cyclosporin A protects against ischemia-reperfusion injury in the brain. Brain Res.

[CR100] Sidransky E, Nalls MA, Aasly JO, Aharon-Peretz J, Annesi G, Barbosa ER (2009). Multicenter analysis of glucocerebrosidase mutations in Parkinson’s disease. N Engl J Med.

[CR101] Song W, Song Y, Kincaid B, Bossy B, Bossy-Wetzel E (2013). Mutant SOD1G93A triggers mitochondrial fragmentation in spinal cord motor neurons: neuroprotection by SIRT3 and PGC-1alpha. Neurobiol Dis.

[CR102] Spencer B, Potkar R, Trejo M, Rockenstein E, Patrick C, Gindi R (2009). Beclin 1 gene transfer activates autophagy and ameliorates the neurodegenerative pathology in alpha-synuclein models of Parkinson’s and Lewy body diseases. J Neurosci.

[CR103] Srivastava S, Diaz F, Iommarini L, Aure K, Lombes A, Moraes CT (2009). PGC-1alpha/beta induced expression partially compensates for respiratory chain defects in cells from patients with mitochondrial disorders. Hum Mol Genet.

[CR104] Strum JC, Shehee R, Virley D, Richardson J, Mattie M, Selley P (2007). Rosiglitazone induces mitochondrial biogenesis in mouse brain. J Alzheimers Dis.

[CR105] Tanaka M, Machida Y, Niu S, Ikeda T, Jana NR, Doi H (2004). Trehalose alleviates polyglutamine-mediated pathology in a mouse model of Huntington disease. Nat Med.

[CR106] Tolkovsky AM (2009). Mitophagy. Biochim Biophys Acta.

[CR107] Tontonoz P, Hu E, Spiegelman BM (1994). Stimulation of adipogenesis in fibroblasts by PPAR gamma 2, a lipid-activated transcription factor. Cell.

[CR108] Viscomi C, Bottani E, Civiletto G, Cerutti R, Moggio M, Fagiolari G (2011). In vivo correction of COX deficiency by activation of the AMPK/PGC-1alpha axis. Cell Metab.

[CR109] Wang QJ, Ding Y, Kohtz DS, Mizushima N, Cristea IM, Rout MP (2006). Induction of autophagy in axonal dystrophy and degeneration. J Neurosci.

[CR110] Wang X, Carlsson Y, Basso E, Zhu C, Rousset CI, Rasola A (2009). Developmental shift of cyclophilin D contribution to hypoxic-ischemic brain injury. J Neurosci.

[CR111] Weydt P, Pineda VV, Torrence AE, Libby RT, Satterfield TF, Lazarowski ER (2006). Thermoregulatory and metabolic defects in Huntington’s disease transgenic mice implicate PGC-1alpha in Huntington’s disease neurodegeneration. Cell Metab.

[CR112] Wong E, Cuervo AM (2010). Autophagy gone awry in neurodegenerative diseases. Nat Neurosci.

[CR113] Wu Y, Li X, Zhu JX, Xie W, Le W, Fan Z (2011). Resveratrol-activated AMPK/SIRT1/autophagy in cellular models of Parkinson’s disease. Neurosignals.

[CR114] Xu S, Zhong M, Zhang L, Wang Y, Zhou Z, Hao Y (2009). Overexpression of Tfam protects mitochondria against beta-amyloid-induced oxidative damage in SH-SY5Y cells. FEBS J.

[CR115] Zhang X, Li L, Chen S, Yang D, Wang Y, Wang Z (2011). Rapamycin treatment augments motor neuron degeneration in SOD1(G93A) mouse model of amyotrophic lateral sclerosis. Autophagy.

[CR116] Zhang K, Shi P, An T, Wang Q, Wang J, Li Z (2013). Food restriction-induced autophagy modulates degradation of mutant SOD1 in an amyotrophic lateral sclerosis mouse model. Brain Res.

[CR117] Zheng B, Liao Z, Locascio JJ, Lesniak KA, Roderick SS, Watt ML (2010). PGC-1alpha, a potential therapeutic target for early intervention in Parkinson’s disease. Sci Transl Med.

[CR118] Zhong N, Xu J (2008). Synergistic activation of the human MnSOD promoter by DJ-1 and PGC-1alpha: regulation by SUMOylation and oxidation. Hum Mol Genet.

[CR119] Zhu J, Wang KZ, Chu CT (2013). After the banquet: mitochondrial biogenesis, mitophagy, and cell survival. Autophagy.

